# Osteoarthritic Milieu Affects Adipose‐Derived Mesenchymal Stromal Cells

**DOI:** 10.1002/jor.24446

**Published:** 2019-08-30

**Authors:** Cristina Manferdini, Francesca Paolella, Elena Gabusi, Luca Cattini, Markus Rojewski, Hubert Schrezenmeier, Olga Addimanda, Riccardo Meliconi, Gina Lisignoli

**Affiliations:** ^1^ IRCCS Istituto Ortopedico Rizzoli SC Laboratorio di Immunoreumatologia e Rigenerazione Tissutale Bologna Italy; ^2^ Institut für Transfusionsmedizin Universität Ulm Ulm Germany; ^3^ Institut für Klinische Transfusionsmedizin und Immungenetik DRK‐Blutspendedienst Baden‐Württemberg–Hessen & Universitätsklinikum Ulm Germany; ^4^ IRCCS Istituto Ortopedico Rizzoli SSD Medicina e Reumatologia Bologna Italy; ^5^ Dipartimento di Scienze Biomediche e neuromotorie Università degli studi di Bologna Bologna Italy

**Keywords:** adipose‐derived mesenchymal stromal cells, cytokine, hypoxia, migration, synovial fluid

## Abstract

The objective of this study was to define the effects of osteoarthritic (OA) milieu on good manufactured practice‐adipose‐derived mesenchymal stromal cells (GMP‐ASC) that are commonly utilized in cell therapies. Two different OA milieu: OA synovial fluid (SF) and OA‐conditioned medium (CM) from synoviocytes were used to treat GMP‐ASC both in normoxia or hypoxia. GMP‐ASC were tested for cell migration, proliferation, cytokine receptors expression (CXCR1, CXCR2, CXCR3, CXCR4, CXCR7, CCR1, CCR2, CCR3, CCR5, IL6R), and cytokines (CXCL8/IL8, CXCL10/IP10, CXCL12/SDF‐1, CCL2/MCP1, CCL3/MIP1α, CCL4/MIP1β, CCL5/RANTES, IL6) release. Healthy SF was used as controls. We demonstrated that GMP‐ASC show an increase in proliferation, migration, and modulation of CXCR1, CXCR3, CCR1, and CCR5 receptors in hypoxic condition. Moreover, GMP‐ASC migration increased 15‐fold when treated either with OA‐SF or OA‐CM compared with healthy SF both in normoxia and hypoxia. GMP‐ASC treated in both OA milieu showed an increase in CXCR3, CCR3, and IL6R and a decrease in CCR1 and CCR2 receptors. In OA‐SF, we detected higher amount of CXCL10/IP10 than in OA‐CM, while CCL2/MCP1 and CCL4/MIP1β were higher in OA‐CM compared with OA‐SF. CXCL10/IP10 was the only chemokine of the OA milieu, which was down‐modulated after treatment with GMP‐ASC. In conclusion, we demonstrated specific effects of OA milieu on both GMP‐ASC proliferation, migration, and cytokine receptor expression that were strictly dependent on the inflammatory and hypoxic environment. The use of characterized OA milieu is crucial to define the therapeutic effect of GMP‐ASC and indicates that CXCL10/IP10–CXCR3 axis is partially involved in the GMP‐ASC effect on synovial macrophages. © 2019 The Authors. *Journal of Orthopaedic Research*® published by Wiley Periodicals, Inc. on behalf of Orthopaedic Research Society. J Orthop Res 38:336‐347, 2020

## Graphical abstract

The objective of this study was to define in vitro the effects of osteoarthritic (OA) milieu and hypoxia on good manufactured practice‐adipose‐derived mesenchymal stromal cells (GMP‐ASC) that are commonly utilized in cell therapies. Hypoxia increased GMP‐ASC proliferation. OA milieu‐treated GMP‐ASC showed an increase in cellular migration, as well as in CXCR3, CCR3, and IL6R and a decrease in CCR1 and CCR2 receptors in hypoxia. CXCL10/IP10 was the only chemokine of the OA milieu down‐modulated after treatment with GMP‐ASC.

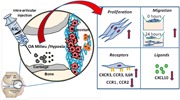

**Abbreviations**
ASC: adipose‐derived mesenchymal stromal cells
BM‐MSC: bone marrow mesenchymal stromal cells
BSA: bovine serum albumin
CCR1: C‐C chemokine receptor type 1
CCR2: C‐C chemokine receptor type 2
CCR3: C‐C chemokine receptor type 3
CCR5: C‐C chemokine receptor type 5
CXCR1: CXC motif chemokine receptor 1
CXCR3: CXC motif chemokine receptor 3
CXCR4: CXC motif chemokine receptor 4
CXCR7: CXC motif chemokine receptor 7
CXCL8/IL‐8: CXC‐chemokine ligand 8/Interleukin 8
CXCL10/IP10: CXC‐chemokine ligand 10/ interferonγ‐induced protein 10
CXCL12/SDF‐1: CXC‐chemokine ligand 12/ stromal cell‐derived factor‐1
CCL2/MCP1: CC‐chemokine ligand 2/monocyte chemotactic protein‐1
CCL3/MIP1α: CC‐chemokine ligand 3/macrophage inflammatory protein‐1α
CCL4/MIP1 β: CC‐chemokine ligand 4/macrophage inflammatory protein‐1 β
CCL5/RANTES: CC‐chemokine ligand5/receptor‐activated normal T‐cell expressed and secreted
CCL11/Eotaxin: CC‐chemokine ligand 11/ eotaxin
CD: clusters of differentiation
CTR: control
FACS: fluorescence‐activated cell sorting
FITC: fluorescein isothiocyanate conjugate
GFP: green fluoresent protein
GMP: good manufacturing practice
Healthy SF: healthy synovial fluid
INFγ: interferon γ
IL1β: interleukin 1β
IL6: interleukin 6
IL6R: interleukin 6 receptor
MFI: median fluorescence intensity
OA: osteoarthritis
OA‐CM: OA‐conditioned medium from synoviocytes
OA‐SF: OA synovial fluid
PBS: phosphate‐buffered saline
PLP: platelet lysate
SC: stromal cell
SD: standard deviation
TNF‐α: tumor necrosis factor α
αMEM: minimum essential medium eagle‐α modification

Adipose‐derived mesenchymal stromal cells (ASC) are promising candidates for cell‐based therapy in osteoarthritis (OA) patients as they exert anti‐inflammatory, immunomodulatory, anti‐fibrotic, and their anti‐hypertrophic effects in the joint tissue.^1^, ^2^, ^3^, ^4^ A successful strategy to counteract OA would consist of long‐term modulation of degenerative joint environment by simultaneously reducing inflammation and promoting tissue regeneration.^5^, ^6^ ASC can be easily harvested from patients by a simple, minimally invasive procedure and are more abundant than the bone marrow mesenchymal stromal cells (BM‐MSC).^7^, ^8^, ^9^ Both stromal cell (SC) types have adipogenic, osteogenic, and chondrogenic differentiation potential and display immunosuppressive properties, both in vitro and in vivo.^9^, ^10^, ^11^

A role of inflammatory signals on SC activation was also suggested in one of our previous studies reporting that in vitro co‐culture of human ASC with high inflamed OA chondrocytes and synoviocytes, were able to downregulate the expression of several inflammatory molecules, while no effects were observed when co‐cultured with low inflamed OA chondrocytes or synoviocytes.^2^, ^12^ This is further supported by recent findings that the protective effect of ASCs was only observed in the mice model of osteoarthritis when high synovial inflammation was present at the time of injection.^13^ In this animal model, green fluorescent protein (GFP)‐labeled ASCs were localized within the synovial lining layer in close contact to synovial macrophages.^14^

Therefore, one of the more interesting characteristics of MSC is their ability to migrate to areas of tissue injury;^15^ however, the exact mechanisms used by ASC, activated by the OA milieu to migrate to target tissues, have not been fully elucidated. Locally injected‐ASC are both affected by the OA synovial fluids, as well as by the soluble factors specifically released by their target synovium tissue. It has been shown that OA synovial fluid mainly contains cytokines (i.e., IL1β, IL6, TNFα) and chemokines (i.e., CXCL8/IL8, CXCL12/SDF‐1, CCL3/MIP1α) mediators.^16^, ^17^, ^18^

The migration and adhesion of injected‐ASC in OA joints will depend mainly on the chemotactic factors secreted by the OA joint tissues and by the expression of receptor for these factors.^19^, ^20^, ^21^ Various papers have evaluated the impact of cytokines and chemokines present in OA synovial fluids on cartilage tissue.^17^, ^22^, ^23^ However, little is known about the OA milieu factors that could enhance the migration and tissue‐specific engraftment of exogenously injected MSC for successful therapeutics in OA.

Different molecules are involved in or necessary for the different steps in the homing process, and chemokines receptors (G‐protein coupled receptors) represent the main group.^15^ It has been extensively demonstrated that the CXCR4–stromal‐derived factor‐1 (CXCL12/SDF‐1) axis is critical for bone marrow homing,^24^, ^25^, ^26^, ^27^ and a number of the other cytokines and growth factors such as CCL2/MCP1, TNFα, CXCL10/IP10 have been shown to increase migration in vitro.^19^, ^28^

Another important environmental factor that affects MSC after OA injection is the oxygen concentration, as it is known that synovial fluid in OA patients is characterized by low oxygen concentration.^29^, ^30^, ^31^ MSC are normally cultured under 20% oxygen tension. To better understand their function in the local environment, it is necessary to evaluate whether MSC also tolerate lower oxygen concentrations (<2%).^32^, ^33^ In fact, it has been demonstrated that hypoxic conditions (1% O_2_) affect MSC function by reducing their senescence, increasing proliferation, and maintaining the differentiation properties.^34^, ^35^ In addition, a hypoxic environment increases the expression of chemokine receptors such as CXCR4 and CXCR7.^36^

A better understanding of mechanisms supporting ASC migration and the identification of cytokine receptors directly involved in this process will allow to clarify the chemotactic capacities of these cells and their therapeutic effects on the target tissues. Therefore, in the present study, both the effect of low oxygen concentration OA synovial fluid (OA‐SF) and OA‐conditioned medium (OA‐CM) from synoviocytes was evaluated on migration properties and cytokine receptors of human ASC to define the factors mainly responsible of these processes.

## METHODS

### Specimens

Subcutaneous abdominal fat was obtained from 10 OA patients undergoing liposuction, when recruited for the ADIPOA2 clinical trial phase IIb European project (Grant agreement number 643809), as previously described.^37^ Knee synovial fluid samples were obtained from 20 OA patients. The specimens were immediately centrifuged at 15,000 rpm for 20 min, and the supernatant was aliquoted and stored at −80°C until the time of analysis. Healthy synovial fluid was acquired from articular engineering (Articular Engineering, Northbrook, IL).

Synovia was obtained from 15 OA patients undergoing total knee replacement surgery.

The study was approved by the Rizzoli Orthopedic Institute Ethical Committee and all patients provided their informed consent (Protocol number LIRT/ADIPOA2).

### Cell Cultures

ASCs were isolated from subcutaneous abdominal fat according to Good Manufacturing Practice (GMP) 38 and grown in minimum essential medium eagle‐α modification (αMEM) (Gibco, Life Technologies, Paisley, UK) supplemented with platelet lysate (PLP). The cell factory characterized, at both passage 0 and 1, the ASC for the expression of classical markers: CD13, CD36, CD73, CD90, and CD105 (BD Biosciences, USA) and the absence of hematopoietic and endothelial markers: CD14, CD31, CD34, CD45 (BD Biosciences).^39^ The number of CFU‐f/10^6^ cells and population doubling were also tested; data were reported in Supplementary Figure S1.

Synoviocytes were isolated as previously reported 12 and conditioned medium of synoviocytes (OA‐CM) was collected 48 h later in serum‐free medium. The experimental design is summarized in Supplementary Figure S2.

### Cytokine Assays

OA and healthy synovial fluids were treated with hyaluronidase (10 mg/ml; Sigma‐Aldrich, Darmstadt, Germany) for 30 min at 37°C. OA‐CM from synoviocytes and supernatant of GMP‐ASC were analyzed for IL6, CXCL8/IL8, CXCL10/IP10, CXCL12/SDF‐1, CCL2/MCP1, CCL3/MIP1α, CCL4/MIP1β, and CCL5/RANTES using multiplex bead‐based sandwich immunoassay kits (Bio‐Rad Laboratories Inc., Segrate, Italy) following the manufacturer's instructions.

### Hypoxic GMP‐ASC Culture

To test hypoxic conditions, 1 × 10^4^ cells were placed inside a chamber from Billups‐Rothenberg, Inc. (San Diego, CA) for 48 h, where a mixture of gas (95% N_2_ and 5% CO_2_) was injected resulting in 1.5% O_2_. The oxygen percentage was controlled by an Oxygen Analyzer (Vascular Technology, Nashua, NH). Pimonidazole hydrochloride (Hypoxyprobe™‐1 kit; Chemicon, Burlington, MA) is a substance with low molecular weight that binds only to cells that have an oxygen tension of 10 mm Hg (pO2~1,2%) or lower, and was used to evaluate hypoxia of ASC.^40^

Cultures were stained with Hypoxyprobe™‐1, according to the manufacturer's protocol. One experiment in triplicate was performed for each time point.

### GMP‐ASC Migration

GMP‐ASC migration assays (shown in Supplementary Figure S3) were performed using 8‐µm pore size HTS transwell polycarbonate insert systems (Corning Incorporated, ME, Kennebunk). Afterwards, 1 × 10^4^ASC were seeded onto the membrane in the upper chambers. The lower chamber was filled with 150 µl serum‐free medium containing: (i) six OA synovial fluids different pools were prepared and used at 1:5 dilution; (ii) different OA‐CM from synoviocytes (*n* = 9) were tested. GMP‐ASC migration was also evaluated using the following cytokines approximately at the mean concentration previously detected in the two OA milieu: CXCL8/IL8 (6,000; 1,500 pg/ml), CXCL10/IP10 (2,500; 12,000 pg/ml), CXCL12/SDF‐1 (100; 150 pg/ml), CCL2/MCP1 (2,200; 400 pg/ml); CCL3/MIP1α (100; 50 pg/ml); CCL4/MIP1β (3,000; 300 pg/ml), CCL5/RANTES (200;400 pg/ml); and IL6 (15000;3000 pg/ml) (R&D Systems, Inc., Minneapolis, MN). Serum‐free medium and 20% fetal bovine serum (FBS) were used as negative (CTR−) and positive (CTR+) controls, respectively.

After 18 h under standard culture condition (1.5% O_2_ and 20% O_2_), cells showing no migration from the upper side of the membrane were removed with a cotton swab. Those cells that migrated through the membrane were stained with Calcein AM (Thermo Fisher Scientific) and finally fixed in 10% formaldehyde. The number of migrating ASC was quantified using fluorescent plate reader Tecan (Tecan Italia S.r.l., Italy). The fluorescence intensity of each experimental condition (in triplicate) was expressed as fold increase versus basal control, considered equal to 1.

### Flow Cytometry

GMP‐ASC alone and treated either with OA‐CM or OA‐SF were characterized by flow cytometry using the following markers CXCR1, CXCR3, CXCR4, CCR1, CCR2, CCR3, CCR5 (R&D), CXCR7 (Abcam, Cambridge, UK), and IL6R (GeneTex Inc., Irvine, CA).

In brief, after harvesting cells upon detachment, they were washed twice with phosphate‐buffered saline (PBS), centrifuged, and washed in a flow cytometry buffer (PBS supplemented with 2% bovine serum albumin [BSA] and 0.1% sodium azide).

Aliquots of 1 × 10^5^ cells were then incubated with primary antibodies at 10 µg/ml 4 °C for 30 min, washed twice with a flow cytometry buffer, and incubated with polyclonal rabbit anti‐mouse and goat anti‐rabbit immunoglobulins/fluorescein isothiocyanate (FITC) conjugate (Dako Cytomation, Glostrup, Denmark) at 4 °C for 30 min. After two final washes, the cells were analyzed using a fluorescence‐activated cell sorting (FACS) CantoII Cytometer (Becton Dickinson). For isotype control, non‐specific mouse IgG was substituted for the primary antibody.

### GMP‐ASC Blocking Experiments for CXCR3/CXCL10/IP10 Axis

GMP‐ASC alone and treated either with OA‐CM or OA‐SF were tested for cytokine, as previously described. Blocking experiments were performed with SCH546738 (MCE MedChemExpress, Monmouth Junction, NJ), a specific CXCR3 receptor antagonist. Three different concentrations (1, 10, and 20 nM) were tested and 10 nM was defined for the blocking experiments. In brief, GMP‐ASC were pre‐treated for 2 h with or without 10 nM SCH546738 and then OA‐SF and OA‐CM were added for 48 h both in normoxia and hypoxia conditions.

### Statistical Analysis

Statistical analysis was performed using non‐parametric tests because the data did not have normal or strongly asymmetric distribution. Wilcoxon's signed rank test was used to compare normoxic versus hypoxic conditions and Kruskal–Wallis and Dunn's post hoc or Mann–Whitney *U* test for unpaired data. CSS Statistica Statistical Software (Statsoft Inc., Tulsa, OK) was used for the analysis and values of *p* < 0.05 were considered statistically significant. Values were expressed either as the median and interquartile range or as mean with 95% confidence interval or as mean ± standard deviation (SD) depending on the distribution.

## RESULTS

### GMP‐ASC Characterization in Normoxic and Hypoxic Conditions

GMP‐ASC grown in normoxic and hypoxic conditions were first characterized analyzing morphological, phenotypical, and proliferative changes.

**Figure 1:**
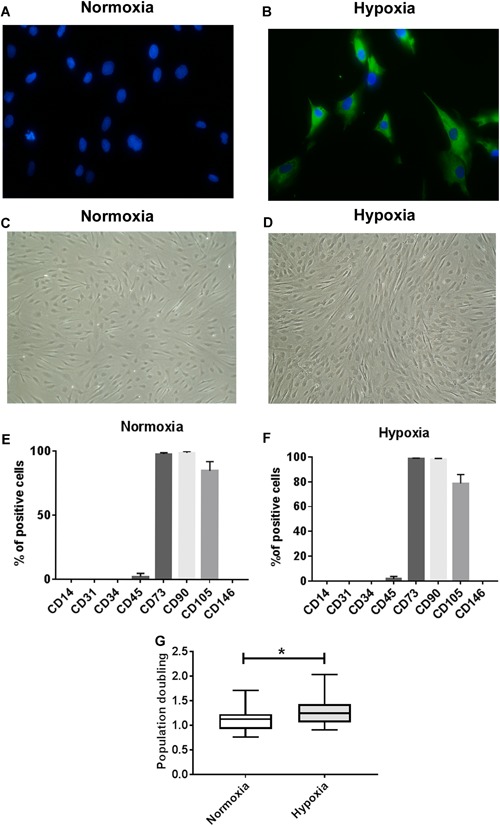
Basal good manufactured practice‐adipose‐derived mesenchymal stromal cells (GMP‐ASC) characterization in normoxia and hypoxia. (A and B) Effect of oxygen deprivation on GMP‐ASC. Representative image of Hypoxyprobe staining in normoxic (A) and hypoxic (B) GMP‐ASC after at 48 h of treatment; pimonidazole stains hypoxic cells (green) and 4’,6‐diamidino‐2‐phenylindole stains all cell nuclei (blue). Scale bars: 50 µm. (C and D) Morphology of GMP‐ASC in normoxic (C) and hypoxic conditions (D), Scale bars: 100 µm. (E and F) Percentage of positive GMP‐ASC markers both in normoxia (E) and hypoxia (F). (G) Population doubling of GMP‐ASC in normoxia and hypoxia. Values are expressed as the mean ± standard deviation. Significant differences *p < 0.05 between normoxia and hypoxia. [Color figure can be viewed at wileyonlinelibrary.com]

GMP‐ASC cells cultured in hypoxic (1.5% O_2_) condition exhibited a significantly increased level of hypoxyprobe staining (Fig. 1 B) compared with cells cultured in normoxic condition (20% O_2_) for 48 h (Fig. 1A). These results indicate that the cells were sensitive to hypoxia under oxygen deprivation conditions. As shown in Figure 1C–D, the cells were all viable and did not show any morphologic changes in both conditions evaluated. Flow cytometric analysis showed a high expression of CD73, CD90, and CD105 and a very low or absent expression of CD14, CD31, CD34, CD45, and CD146 in both conditions (Fig. 1E–F). Interestingly, as shown in Figure 1G, it was observed that GMP‐ASC proliferation (*p* = 0.0039) signiﬁcantly increased in hypoxic condition compared with normoxic.

### OA Mileu Released Factors

We then evaluated different factors directly involved in GMP‐ASC chemotaxis, both in OA‐CM from synoviocytes and OA synovial fluid that represent the target organ and the microenvironment, respectively. Moreover, we tested healthy synovial fluid as control milieu. As shown in Table 1, CXCL12/SDF‐1, CCL3/MIP1α, CCL5/RANTES, and CCL11/Eotaxin were detected in the same amount both in OA‐SF and OA‐CM. CXCL10/IP10 was produced in higher amounts in OA‐SF compared with OA‐CM and CXCL8/IL8, CCL2/MCP1, and CCL4/MIP1β were higher in OA‐CM compared with OA‐SF. In healthy‐SF, all the factors analyzed were produced in the same (CXCL8/IL8, CXCL12/SDF‐1, CCL2/MCP1, CCL11/Eotaxin) or significantly lower (CXCL10/IP10, CCL3/MIP1α, CCL4/MIP1β, CCL5/RANTES, and IL6) amount compared with OA‐SF. The same differences were found between healthy‐SF and OA‐CM, except for CXCL10/IP10 that was higher in healthy SF.

**Table 1:** Cytokines Detected in Osteoarthritis‐Conditioned Medium (OA‐CM) Synoviocytes, OA Synovial Fluid (SF), and Healthy Synovial Fluid

Biomarkers	OA‐CM	OA‐SF	Healthy SF
CXCL8/IL8	5924.45 ± 5421.06	1274.45 ± 766.70	1059.66 ± 609.45
CXCL10 /IP10	2644.45 ± 2034.52*, ^***^	12239.54 ± 7935.60*, ^**^	4404.2 ± 1376.40^**^, ^***^
CXCL12/SDF‐1	103.86 ± 10.38	128.75 ± 45.26	96.04 ± 17.04
CCL2/MCP1	2210.64 ± 1006.10*, ^***^	387.92 ± 391.76*	180.44 ± 56.66^***^
CCL3/MIP1α	95.92 ± 73.76^***^	45.04 ± 11.43^**^	4.84 ± 1.07^**^, ^***^
CCL4/MIP1β	2933.08 ± 1776.35*, ^***^	302.10 ± 116.86*, ^**^	119.11 ± 50.51^**^, ^***^
CCL5/ RANTES	191.64 ± 99.67^***^	372.34 ± 530.10^**^	5.6 ± 1.96^**^, ^***^
CCL11/Eotaxin	213.9 ± 38.04	334.45 ± 193.17	220.25 ± 154.47
IL6	14283.71 ± 4354.40*, ^***^	2843.53 ± 1351.22*, ^**^	173.15 ± 72.43^**^, ^***^

All data (pg/ml) were expressed as mean ± standard deviation.

* Significant differences between OA‐CM and OA‐SF (*p* < 0.05).

^**^ Significant differences between OA‐SF and healthy SF (p < 0.05).

^***^ Significant differences between OA‐CM and healthy SF (*p* < 0.05).

### Basal GMP‐ASC Released Factors in Normoxic and Hypoxic Conditions

**Figure 2:**
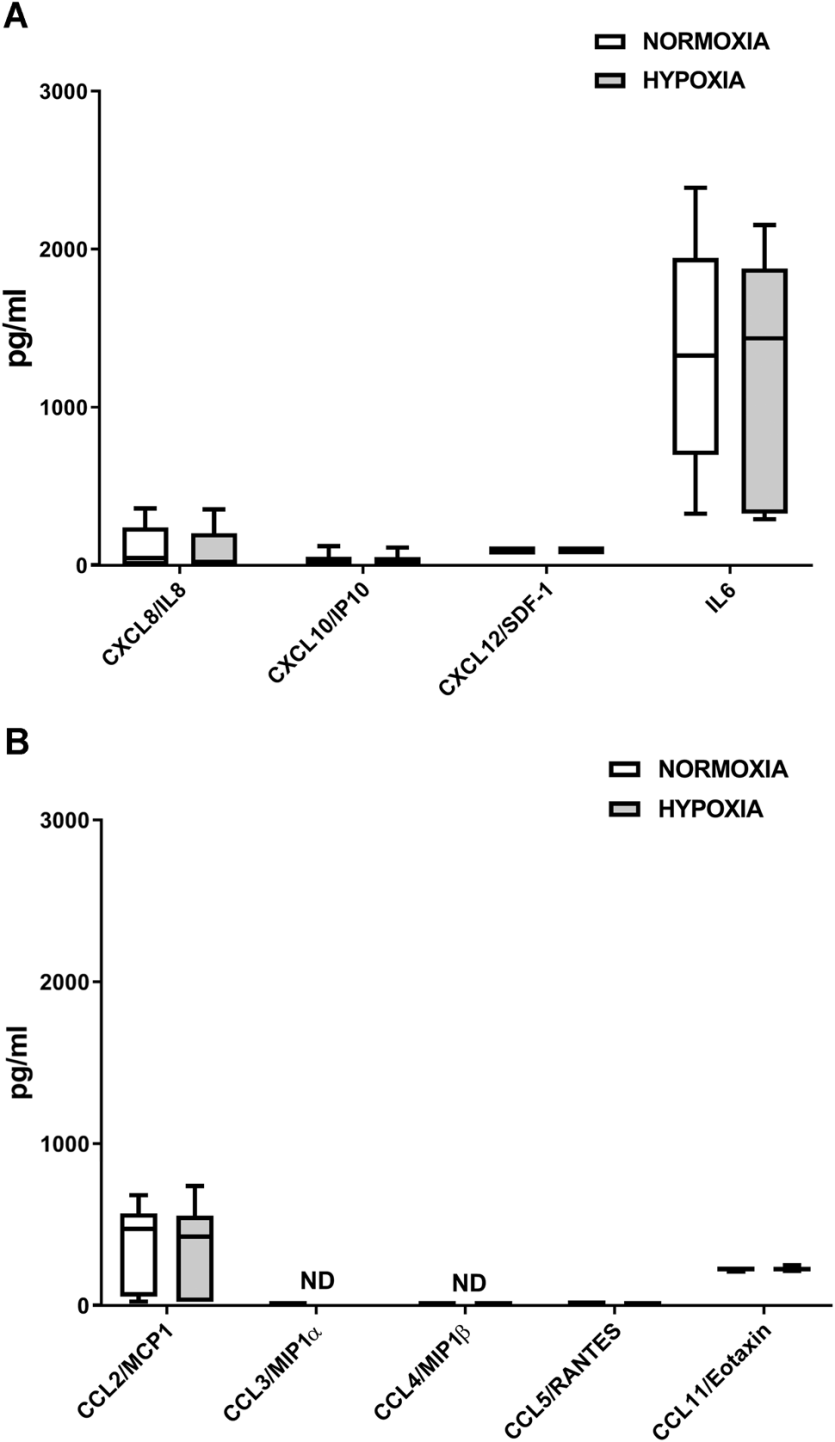
Basal good manufactured practice‐adipose‐derived mesenchymal stromal cells (GMP‐ASC) release of cytokines in normoxia and hypoxia. (A) Basal GMP‐ASC release of CXCL8/IL8, CXCL10/IP10, CXCL12/SDF‐1, and IL6 after 48 h of culture both in normoxia and hypoxia. (B) Basal GMP‐ASC release of CCL2/MCP1, CCL3/MIP1α, CCL4/MIP1β, CCL5/RANTES, CCL11/Eotaxin after 48 h of culture both in normoxia and hypoxia. Data are expressed as median with interquartile range. ND, not detected.

We then tested in basal GMP‐ASC supernatants all the factors previously evaluated in OA milieu in both normoxic and hypoxic conditions. As shown in Figure 2, we found that GMP‐ASC released low amounts of CXCL8/IL8, CXCL10/IP10, CXCL12/SDF‐1, and CCL11/Eotaxin and higher amounts of CCL2/MCP1 and IL6, which were not modulated by hypoxic condition. Interestingly, we did not detect CCL3/MIP1α, CCL4/MIP1β, and very low amount of CCL5/RANTES in GMP‐ASC supernatant.

### GMP‐ASC Migration in OA Milieu in Normoxic and Hypoxic Conditions

**Figure 3:**
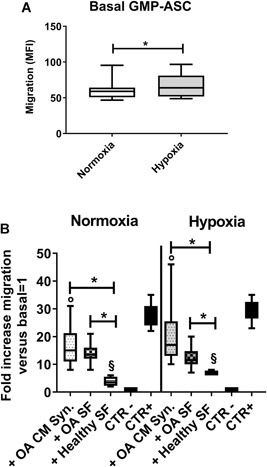
Good manufactured practice‐adipose‐derived mesenchymal stromal cells (GMP‐ASC) migration in normoxia and hypoxia. (A) Basal GMP‐ASC migration both in normoxia and hypoxia. Data are expressed as median fluorescence intensity (MFI). (B) GMP‐ASC migration after treatment for 48 h with OA‐CM (+OA‐CM), OA‐SF (+OA‐SF), healthy synovial fluid (+Healthy‐SF), serum‐free medium (CTR−, negative control) and medium with 20% fetal bovine serum (CTR+, positive control) both in normoxia and hypoxia. Data were calculated as fold increase migration versus basal = 1. Data are expressed as median with interquartile range. Significant differences *p < 0.05 between normoxia and hypoxia. Significant difference was found between normoxia and hypoxia on GMP‐ASC treated with OA‐CM °*p* < 0.05 or with healthy SF ^§^ *p* < 0.05.

To define the effects of OA milieu, we evaluated both in normoxic and hypoxic conditions GMP‐ASC in basal condition and treated either with OA‐CM or OA‐SF and healthy‐SF, as control. As shown in Figure 3A, we found that basal GMP‐ASC in hypoxia showed a significant higher migration than in normoxia (*p* = 0.019). Moreover, when GMP‐ASC were treated either with OA‐CM or healthy‐SF (Fig. 3B), a significant higher migration in hypoxic condition was observed (°*p* = 0.006, ^§^ *p* = 0.049, respectively), but no differences were found using OA‐SF. Interestingly, as shown in Figure 3B, we observed that all treatments approximately increased GMP‐ASC migration 18‐,14‐ and 5‐folds, respectively, compared with basal GMP‐ASC (considered equal to 1). In particular, we evidenced that migration was significantly higher in GMP‐ASC treated either with OA‐CM or OA‐SF than with healthy‐SF, both in normoxic (*p = 0.002, *p = 0.007, respectively) and hypoxic conditions (*p = 0.0007, *p = 0.03, respectively).

### Cytokines Responsible for GMP‐ASC Migration

**Figure 4:**
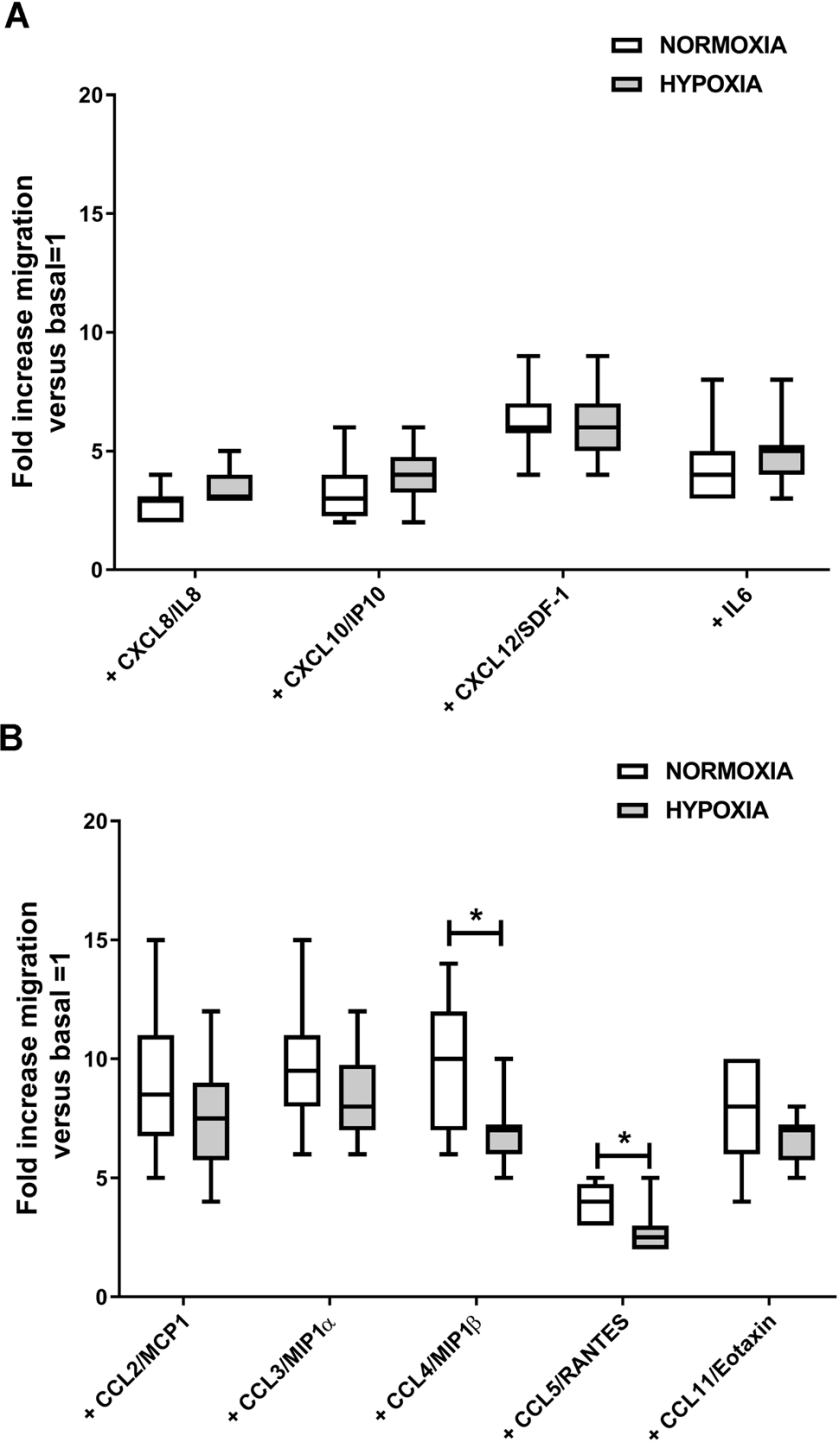
Good manufactured practice‐adipose‐derived mesenchymal stromal cells (GMP‐ASC) migration in normoxia and hypoxia after treatment with cytokines. (A) Basal GMP‐ASC treated with CXCL8/IL8 (+CXCL8/IL8), CXCL10/IP10 (+CXCL10/IP10), CXCL12/SDF‐1 (+CXCL12/SDF‐1), and IL6 (+IL6) for 48 h of culture both in normoxia and hypoxia. (B) Basal GMP‐ASC treated with CCL2/MCP1 (+CCL2/MCP1), CCL3/MIP1α (+CCL3/MIP1α), CCL4/MIP1β (+CCL4/MIP1β), CCL5/RANTES (+CCL5/RANTES), CCL11/Eotaxin (+CCL11/Eotaxin) for 18 h of culture both in normoxia and hypoxia. Data were calculated as fold increase migration versus basal = 1. Data are expressed as median with interquartile range. Significant differences *p < 0.05 between normoxia and hypoxia.

To define which cytokines detected in OA milieu were responsible for preferentially induced GMP‐ASC migration, we tested the effect of single CXCL8/IL8, CXCL10/IP10, CXCL12/SDF‐1, CCL2/MCP1, CCL3/MIP1α, CCL4/MIP1β, CCL5/RANTES, CCL11/Eotaxin, and IL6 previously detected in OA‐SF and OA‐CM. Both the lower and higher concentration of each cytokine detected in OA‐CM and OA‐SF were tested; however, no significant differences in migration were found. Therefore, we used the mean concentration of each cytokine. As shown in Figure 4A, we found that both in normoxic and hypoxic conditions, the treatment with CXCL12/SDF‐1 was more effective in inducing GMP‐ASC migration (fold increase higher than 5) compared with CXCL8/IL8, CXCL10/IP10, and IL6. Moreover, as shown in Figure 4B, we found CCL2/MCP1, CCL3/MIP1α, CCL4/MIP1β, and CCL11/Eotaxin were also able to induce GMP‐ASC migration more than fivefold compared with CCL5/RANTES. However, both in normoxic and hypoxic conditions, GMP‐ASC migration did not change after treatment with CXCL8/IL8, CXCL10/IP10, CXCL12/SDF‐1, CCL2/MCP1, CCL3/MIP1α, CCL11/Eotaxin, and IL6. In contrast, as shown in Figure 4B, we observed GMP‐ASC treated with CCL4/MIP1β or CCL5/RANTES showed a significant lower percentage of migration in hypoxic than in normoxic condition (*p = 0.0069 and *p = 0.035, respectively).

### GMP‐ASC Cytokine Receptors Modulation by OA Milieu in Normoxic and Hypoxic Conditions

**Figure 5:**
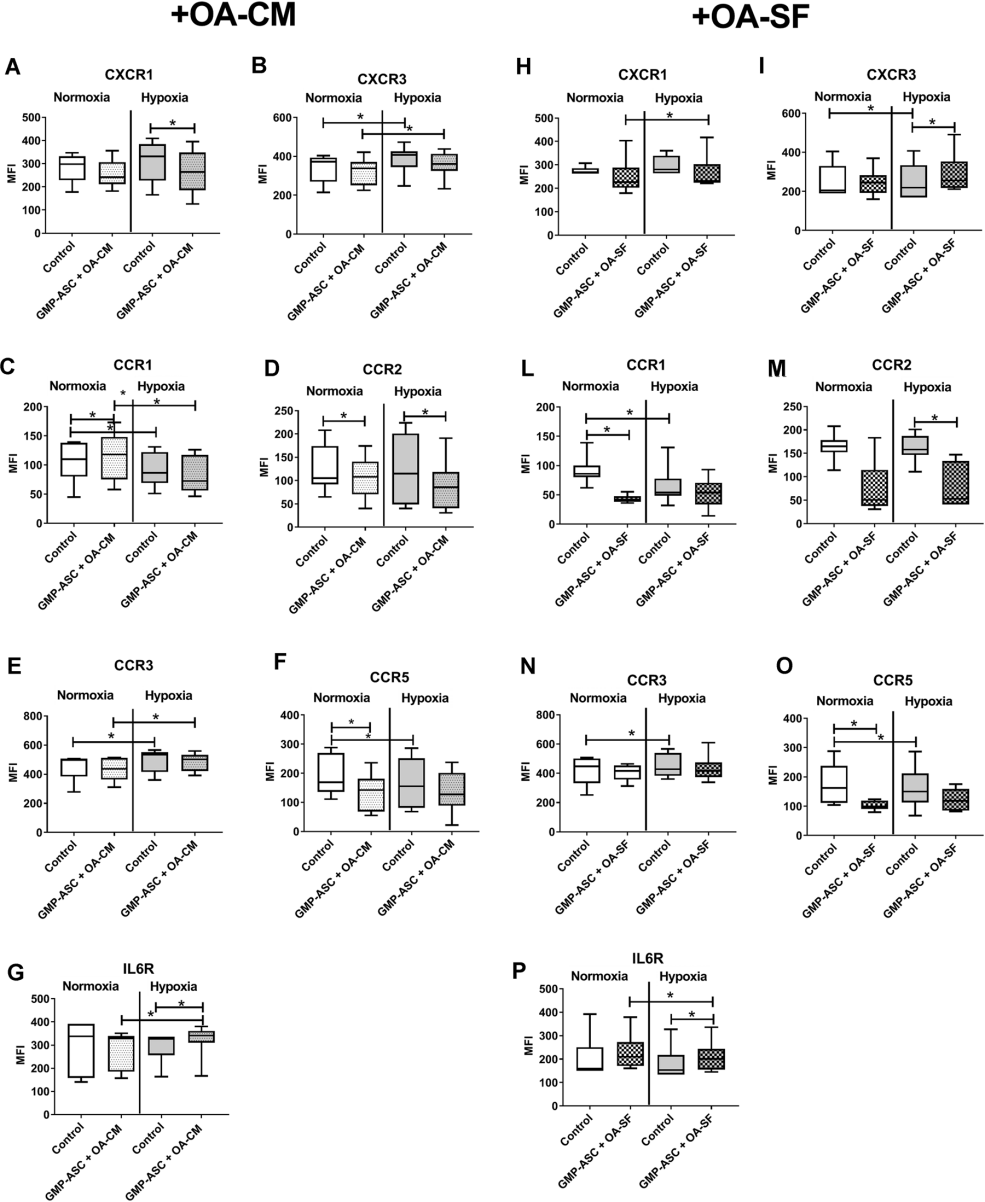
Good manufactured practice‐adipose‐derived mesenchymal stromal cells (GMP‐ASC) expression of cytokine receptors. CXCR1, CXCR3, CCR1, CCR2, CCR3, CCR5, and IL6R receptors expressed on basal GMP‐ASC (Control) or after treatment for 48 h with: (A–G) OA‐CM (GMP‐ASC + OA‐CM), (H–P) OA‐SF (GMP‐ASC + OA‐SF), both in normoxia and hypoxia. Data are expressed as median with interquartile range. Significant differences *p < 0.05 between normoxia and hypoxia. MFI, median fluorescence intensity.

We then defined the effects of OA milieu represented by OA‐CM and OA‐SF on GMP‐ASC cytokine receptors in both normoxic and hypoxic conditions. As shown in Supplementary Table S1, basal GMP‐ASC showed a higher percentage ( >60%) of CXCR1, CXCR3, CXCR4, CXCR7, CCR3, CCR5, and IL6R and a lower percentage ( <40%) of CCR1 and CCR2. We found that only CXCR3 (*p* = 0.046), CCR3 (*p* = 0.046), and CCR5 (*p* = 0.046) percentage were modulated on basal GMP‐ASC in hypoxic compared with normoxic condition. In contrast, GMP‐ASC treated with OA‐CM showed a significant decrease of only the percentage of CCR1 (*p* = 0.017) in hypoxic compared with normoxic condition. Moreover, as shown in Supplementary Table S1, we found in hypoxic condition a significant decrease in the percentage of CCR2 (*p* = 0.02) on GMP‐ASC treated with OA‐CM compared with basal GMP‐ASC, while in normoxic condition, we found a significant decrease of the percentage of CCR5 (*p* = 0.007). GMP‐ASC treated with OA‐SF showed a significant down modulation of CCR1 (*p* = 0.046) and CCR2 (*p* = 0.046) percentage both in normoxia and hypoxia. Afterwards, we evaluated the cytokine receptors density on the cell membrane by evaluating the median fluorescence intensity (MFI). As shown in Figure 5A–G, on basal GMP‐ASC we found a significant increase of CXCR3 (*p* = 0.0085) and CCR3 (*p* = 0.0022) as well as a significant decrease of CCR1(*p* = 0.046) and CCR5 (*p* = 0.0347) in hypoxic compared with normoxic condition. Moreover, CCR2, CXCR1, and IL6R were not modulated, as found for CXCR4 and CXCR7 (data not shown). In contrast, GMP‐ASC treated with OA‐CM showed a significant increase of CXCR3 (*p* = 0.0234), CCR3 (*p* = 0.0008), and IL6R (*p* = 0.00313), and a significant decrease of CCR1 (*p* = 0.0391) in hypoxic compared with normoxic condition. In addition, we found that OA‐CM treatment only in normoxic condition significantly increased CCR1 (*p* = 0.0469), while significantly decreased CCR2 (*p* = 0.0273) and CCR5 (*p* = 0.0039). In contrast, we found that OA‐CM treatment only in hypoxic condition significantly decreased CXCR1 (*p* = 0.0234) and increased IL6R (*p* = 0.0156). CXCR4 and CXCR7 were not modulated by OA‐CM (data not shown).

GMP‐ASC treated with OA‐SF (Fig. 5H–P) showed a significant increase for CXCR1 (*p* = 0.032) and IL6R (*p* = 0.032) in hypoxic compared with normoxic condition. Moreover, we found that OA‐SF treatment only in normoxic condition significantly decreased CCR1 (*p* = 0.032) and CCR5 (*p* = 0.032). In contrast, in hypoxic condition we observed a decrease trend for CXCR1, significant for CCR2 (*p* = 0.032) and an increase for CXCR3 (*p* = 0.032) and IL6R (*p* = 0.032) on GMP‐ASC treated with OA‐SF.

### CXCL10/IP10 Detected in OA Milieu is Modulated by GMP‐ASC Both in Normoxic and Hypoxic Conditions

**Figure 6:**
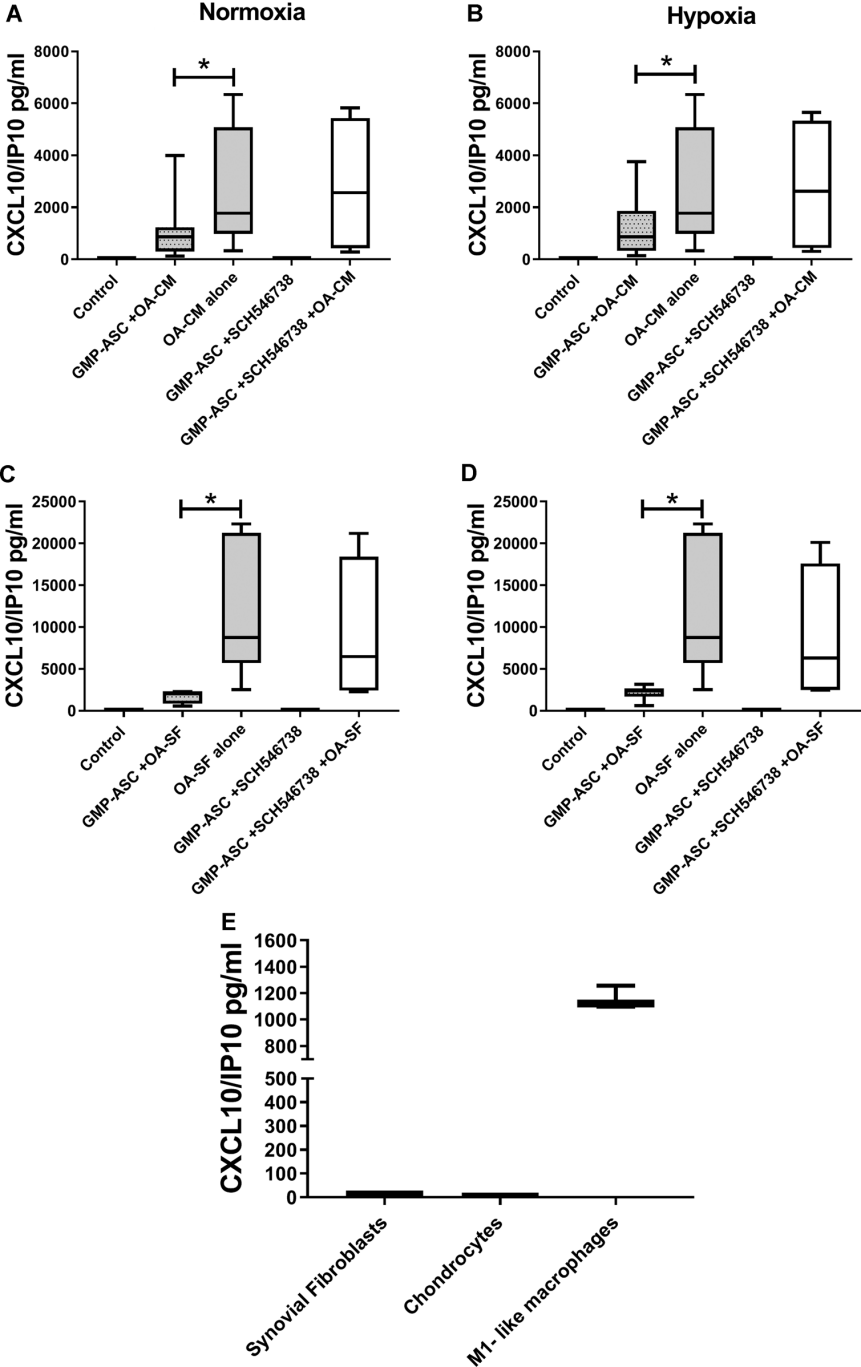
Good manufactured practice‐adipose‐derived mesenchymal stromal cells (GMP‐ASC) modulation of CXCL10/IP10 in normoxia and hypoxia. (A–D) CXCL10/IP10 detected in osteoarthritic (OA) milieu, released by basal GMP‐ASC (Control) or after treatment for 48 h with OA‐CM (GMP‐ASC + OA‐CM) (A, C) or with OA‐SF (GMP‐ASC + OA‐SF) (B and D) with or without SCH546738 CXCR3 receptor antagonist, both in normoxia and hypoxia. Data are expressed as median with interquartile range. Significant differences *p < 0.05. (E) Basal release of CXCL10/IP10 in OA synovial fibroblast alone, chondrocyte alone and M1‐like macrophages alone.

Finally, we evaluated if OA milieu cytokines detected in both OA‐CM or OA‐SF were modulated after culture treatment of GMP‐ASC. To this end, we found that CXCL8/IL8, CXCL12/SDF‐1, CCL2/MCP1, CCL3/MIP1α, CCL4/MIP1β, CCL5/RANTES, CCL11/Eotaxin, and IL6 were not modulated (data not shown), while as shown in Figure 6A–D, CXCL10/IP10 was significantly decreased by OA‐CM and OA‐SF both in normoxic (*p* = 0.003) and hypoxic (*p* = 0.003) conditions. After blocking of CXCL10/IP10 receptor (CXCR3) with the specific antagonist SCH546738, we confirmed that GMP‐ASC effect on CXCL10/IP10 was specific as we did not find CXCL10/IP10 modulated. Moreover, to confirm that CXCL10/IP10 detected in OA‐CM was produced by macrophages, we evaluated this chemokine also on supernatant obtained from previously characterized OA synovial fibroblasts alone, OA isolated chondrocytes, and activated M1‐like macrophages. As shown in Figure 6E, only activated M1‐like macrophages released CXCL10/IP10.

## DISCUSSION

MSC are immune privileged cells widely used for therapeutic purposes due to their anti‐inflammatory potential and capacity to stimulate endogenous cartilage regeneration.^2^, ^3^, ^14^, ^41^ To better elucidate their function, it is fundamental to understand the mechanism that direct ASC homing and engraftment, both to their target organ and specific cell type. It is established that cell chemotaxis is mainly dependent on chemokines that interact with specific chemokine receptors.^15^ It has been shown, both in vitro and in vivo OA models, that the main target of ASC are synovial macrophages.^12^, ^13^, ^41^, ^42^ It is well known that both inflamed OA milieu and hypoxia could affect ASC characteristics. To understand the role of OA milieu and hypoxic condition in modulating and targeting ASC effects, we evaluated ASC migration properties and cytokine receptors that represent two critical points for their therapeutic effects.

First, we evaluated cytokines both in OA‐SF and OA‐CM, as they represent the specific microenvironment and soluble factors released by ASC‐targeted tissue, respectively, encountered by the cells after their joint injection. We evidenced that CXCL12/SDF‐1, CCL3/MIP1α, CCL5/RANTES, and CCL11/Eotaxin were detected in the same amount both in OA‐CM from synoviocytes and synovial fluids, all factors identified as chemoattractants for cells positive for either CXCR4 or CXCR7 or CCR1 or CCR3 or CCR5. Moreover, we found a higher amount of CCL2/MCP1, CCL4/MIP1β, and IL6 in OA‐CM than in OA and healthy synovial fluid. Interestingly, in line with previous papers,^43^, ^44^ a higher amount of CXCL10/IP10 in OA synovial fluid was detected, which is well known to be responsible for the recruitment/activation of immune cells in several organs during homeostatic and inflammatory conditions.^45^ In the synovial fluid, all these factors are released in the joint mainly by cartilage, synovia, immune cells, and Hoffa adipose tissues and contribute to cell migration and homing.^17^

Moreover, hypoxic condition significantly increased ASC proliferation as well as migration capacity, in line with previous studies 28, 46, 47 that also demonstrated an increase of MSC proliferation and migration rate.

Interestingly, when ASC were treated with OA‐CM or OA‐SF we found a significant higher increase of the migration, both in normoxic and hypoxic conditions. Our data was in contrast with Leijs et al.^21^ who found that both inflammatory stimulus (TNFα + IFNγ) and hypoxic condition on the bone marrow MSC showed a decreased cell migration. This contrasting data could be due to the specific inflammatory cocktail they used, which does not adequately represent the OA milieu. In our study, GMP‐ASC migration was also significantly higher in the two OA milieu conditions tested than those treated with healthy synovial fluid, confirming that the higher concentration of factors detected in OA milieu were directly involved in this process. Moreover, using the same mean amount of all the factors detected in OA milieu, we demonstrated more than a fivefold higher increase of GMP‐ASC migration when treated with typical macrophages chemokines such as CCL2/MCP1, CCL3/MIP1α, CCL4/MIP1β, and CCL11/Eotaxin or CXCL12/SDF‐1, which are well known to mediate the migration in *ex vivo*‐expanded MSC.^26^, ^28^, ^48^ All the other factors tested, induced the GMP‐ASC migration; however, with a fold increase lower than five. Hypoxic condition significantly reduced the migration of GMP‐ASC treated with CCL4/MIP1β and CCL5/RANTES, a mechanism that was not observed when using the OA‐CM or OA‐SF. This suggests that the contemporary presence of the cytokines in the OA milieu probably leads to a different chemical interaction among the factors and, consequently, a different effect. These results contribute to the evidence that the GMP‐ASC injection environment is fundamental to consider for defining ASC migration properties. For these reasons, we compared both OA synovial fluid and OA‐CM from synoviocytes as two different OA milieu and we confirmed that they both induced the same increase of GMP‐ASC migration trend in both normoxic and hypoxic conditions.

The interaction between chemotactic factors and their receptors on the surface of transplanted cells is necessary and guides their migration. In our study we found that GMP‐ASC expressed all the receptors tested in normoxic condition and CXCR3, CCR2, and CCR3 were significantly increased in hypoxic condition. This shows that the increase of basal GMP‐ASC migration observed in hypoxic condition could be partially dependent on the high percentage and intensity of the receptors on the cells associated to the higher number of chemokines (i.e., CXCL10/IP10 detected in OA‐SF). In contrast to other studies, we found a high expression of CXCR4/CXCR7 (approximately 98% positive cells) on expanded GMP‐ASC not modulated by hypoxic condition, as reported by other authors.^36^, ^46^, ^49^

In normoxic condition, we evidenced a decrease of CCR2 on GMP‐ASC treated with OA‐CM, both as percentage and intensity of the receptor associated with a decreased CCR5.

Furthermore, we evidenced GMP‐ASC treated with OA‐CM showed a decrease of CCR2 receptor associated with a decreased intensity of CCR1 and an increase of CXCR3, CCR3, and IL6R receptors in hypoxic condition. This trend was also confirmed using OA‐SF, suggesting the OA milieu and hypoxic condition‐induced GMP‐ASC migration, which could be mainly dependent on their specific ligands, CXCL10/IP10, CCL5/RANTES, CCL11/Eotaxin, and IL6 than by CXCL12/SDF‐1. A significant reduction of CXCL10/IP10 in the supernatant of GMP‐ASC treated with OA milieu suggests a direct involvement of the CXCL10/IP10–CXCR3 axis, as confirmed by blocking experiments, which has been recently reported as crucial axis in the severity of OA^50^ by regulating neutrophil‐NK cell cross‐talk.^45^ Indeed, CXCL10/IP10 was highly expressed 45, 51 by synovial macrophages, indicating that GMP‐ASC recruitment and homing to synovial macrophages, as previously demonstrated in OA animal model,^13^ could be partially dependent on CXCL10/IP10–CXCR3 axis.

In conclusion, our study demonstrates a novel specific effect of OA milieu on both GMP‐ASC migration and cytokine receptor expression that were strictly dependent on an inflammatory environment and hypoxic condition. Therefore, only the use of a specific culture OA environment allows to specifically define the real therapeutic effect of GMP‐ASC, suggesting that their specific recruitment to synovial macrophages is partially dependent on CXCL10/IP10–CXCR3 axis. To our knowledge, this is the first study of its kind that focuses on an adequate GMP‐ASC injection environment, presenting novel indications that contribute to understand the role of OA milieu on GMP‐ASC.

## AUTHORS’ CONTRIBUTION

C.M. and G.L. conceived and designed the study; M.R. and H.S. carried out GMP‐clinical grade ASC manufacturing and characterization of ASC; R.M. and O.A. provided the SF samples; C.M., F.P., E.G., and L.C. performed the experiments; C.M., F.P., and L.C. collected and acquired data; C.M., F.P., and G.L. interpreted data and drafted the article; G.L., C.M., F.P., E.G., M.R., H.S., R.M., and O.A. critically revised the manuscript. All the authors approved the final version of the manuscript.

## Supporting information

Supplementary information.

Supplementary information.

Supplementary information.

Supplementary information.
