# Supplementary figures and images for: Osteoarthritic Milieu Affects Adipose‐Derived Mesenchymal Stromal Cells
Source: J Orthop Res. 2019 Aug 30;38(2):336–47. doi: 10.1002/jor.24446 (PMC7003792; doi:10.1002/jor.24446)

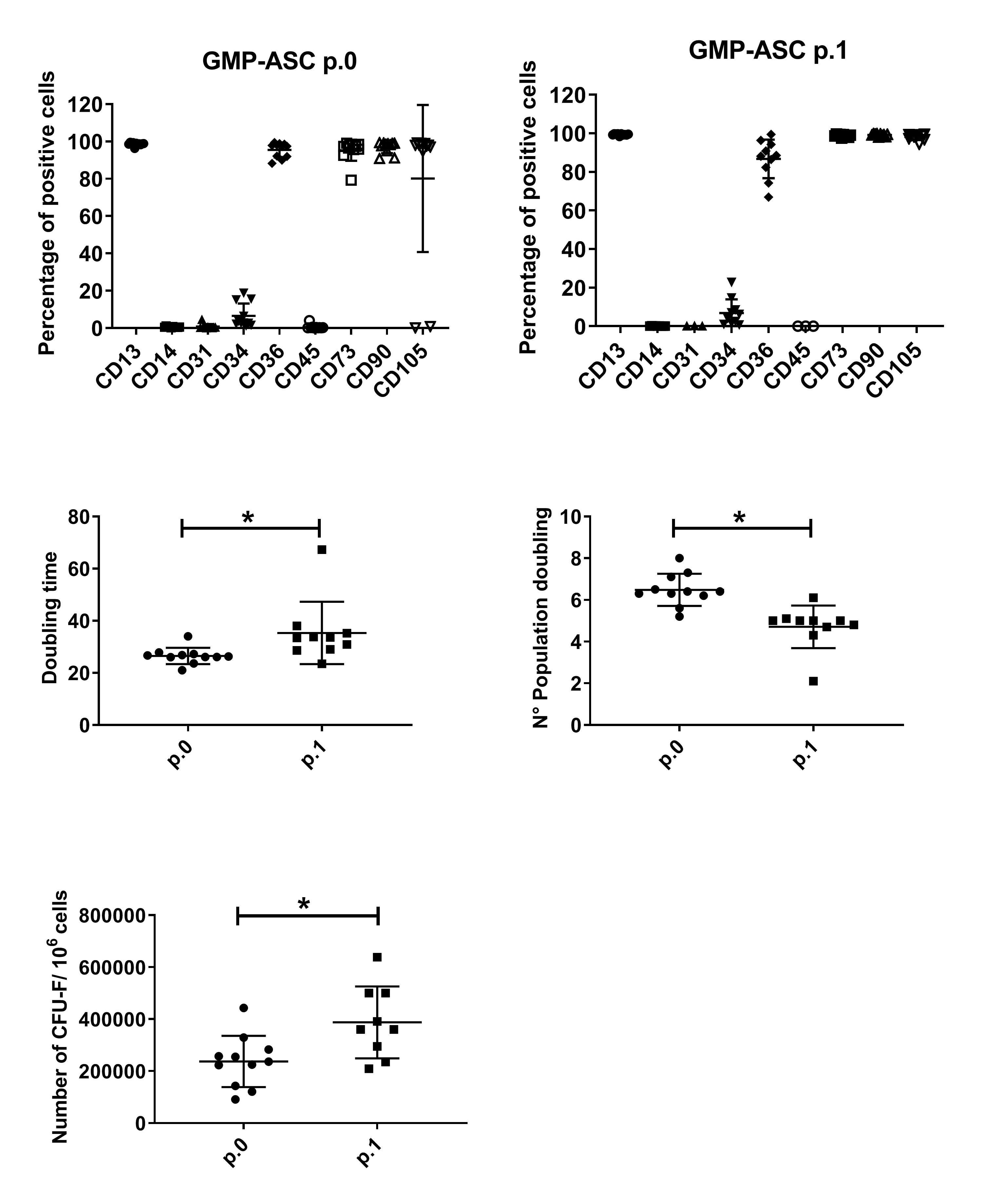

Supplement: Supplementary file 1 — Supplementary information. [file JOR-38-336-s001.tif]

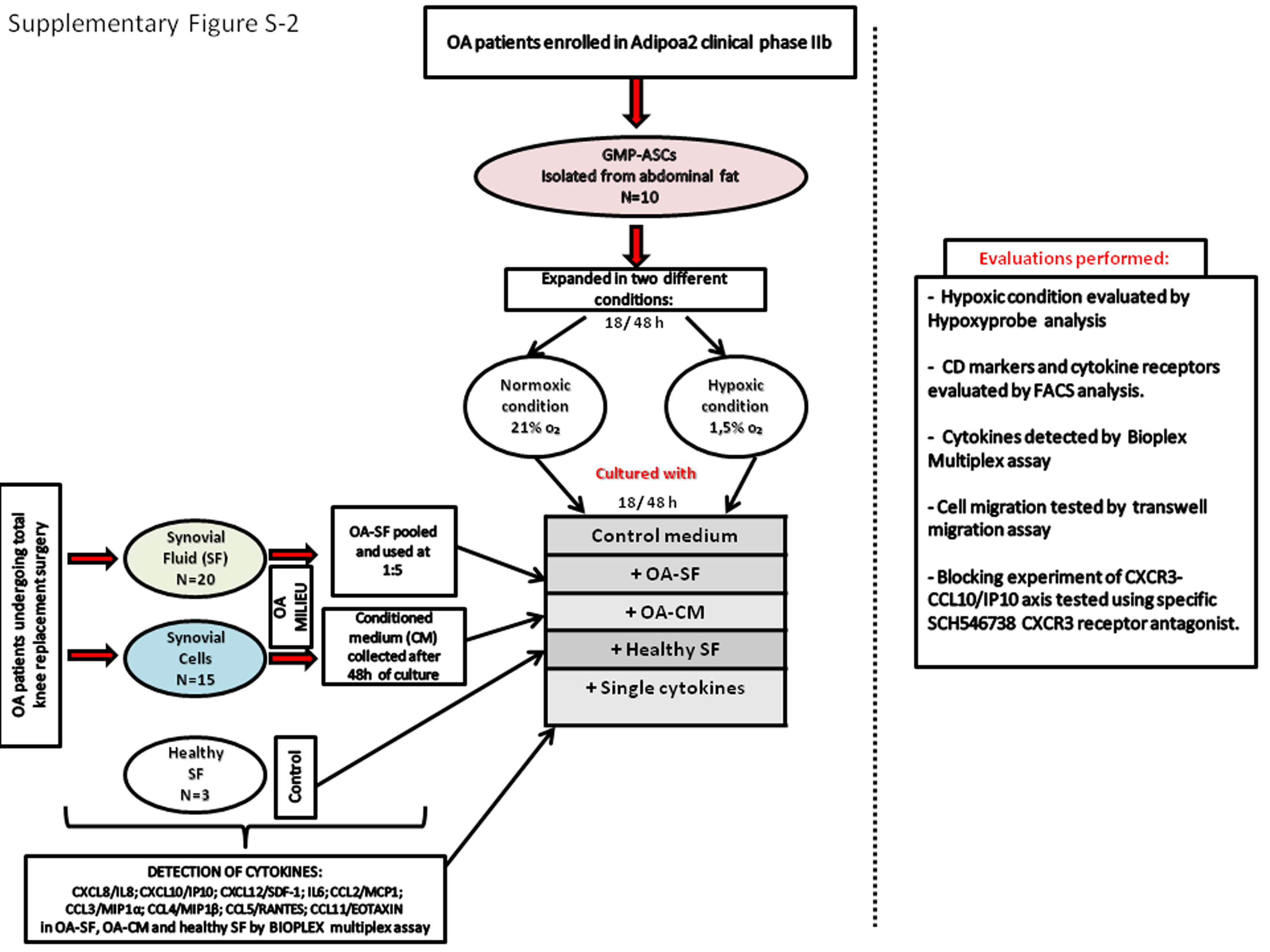

Supplement: Supplementary file 2 — Supplementary information. [file JOR-38-336-s002.tif]

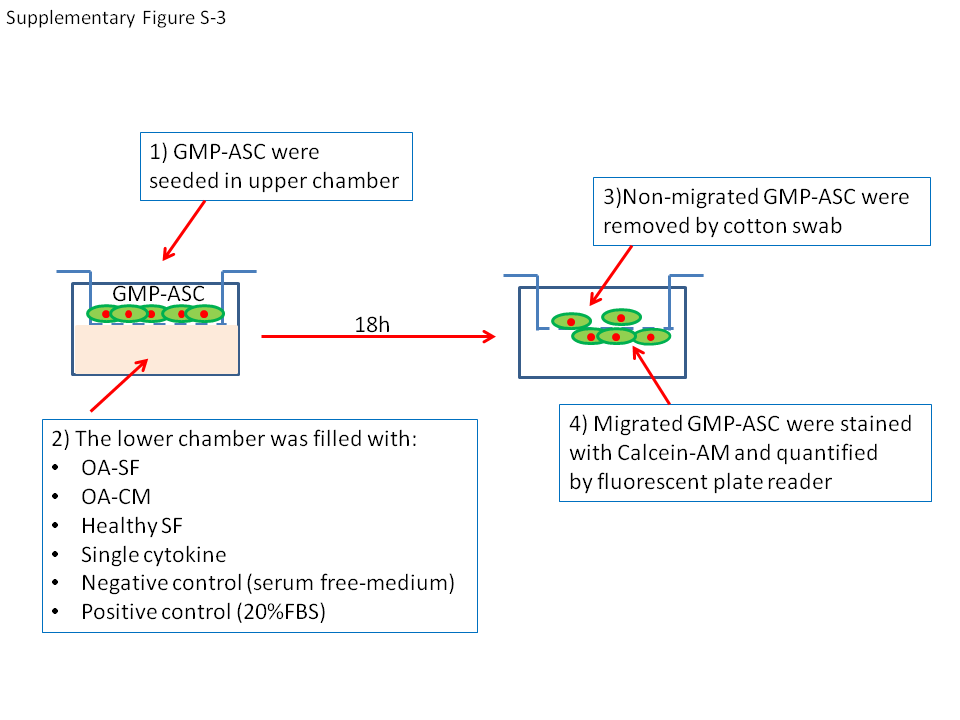

Supplement: Supplementary file 3 — Supplementary information. [file JOR-38-336-s003.tif]
