# Supplementary material for: Osteoarthritic Milieu Affects Adipose‐Derived Mesenchymal Stromal Cells
Source: J Orthop Res. 2019 Aug 30;38(2):336–47. doi: 10.1002/jor.24446 (PMC7003792; doi:10.1002/jor.24446)
Supplement: Supplementary file 4 — Supplementary information. [file JOR-38-336-s004.docx]

**Supplementary Table S-1. Percentage of receptor expression on basal GMP-ASC (Control) and GMP-ASC treated with conditioned medium from OA synoviocyte (GMP-ASC+OA-CM) and OA synovial fluid (GMP-ASC+OA-SF**)**.**

|  | **Normoxia Control** | **Hypoxia**  **Control** | **Normoxia**  **GMP-ASC**  **+ OA-CM** | **Hypoxia**  **GMP-ASC**  **+ OA-CM** | **Normoxia**  **GMP-ASC**  **+ OA-SF** | **Hypoxia**  **GMP-ASC**  **+OA-SF** |
| --- | --- | --- | --- | --- | --- | --- |
| **CXCR1** | 87 ±13 | 92 ±17 | 87±13 | 82±23 | 86±10 | 92±4 |
| **CXCR3** | 85 ±17**^+^** | 94 ±8**^+^** | 97 ±2 | 99 ±1 | 81±18 | 92±6 |
| **CXCR4** | 92 ±13 | 96 ±5 | 94 ±9 | 96 ±5 | 95±2 | 96±1 |
| **CXCR7** | 96 ±7 | 99 ±1 | 98 ±3 | 99 ±2 | 99±1 | 100 |
| **CCR1** | 21 ±12° | 17 ±10# | 26 ±19**^§^** | 16 ±15**^§^** | 8±3° | 10±6# |
| **CCR2** | 33±26° | 41 ±31# ^ | 29 ±20 | 23 ±21 ^ | 24±25° | 19±18# |
| **CCR3** | 95 ±8**^+^** | 99 ±0.5**^+^** | 99 ±1 | 100 | 98±1 | 99±0.5 |
| **CCR5** | 60 ±28 **^+^*** | 46 ±23**^+^** | 42 ±28 * | 43 ±30 | 35±24 | 38±18 |
| **IL6R** | 87±18 | 91 ±7 | 91 ±22 | 98±3 | 86±7 | 93±6 |

All data were expressed as mean percentage of receptor expression ± SD. Wilcoxon matched paired test was used for comparing groups.

**^+^** p= 0.046, Normoxic Control vs Hypoxic Control

* p= 0.007, Normoxic Control vs Normoxic GMP-ASC+OA-CM

**°** p= 0.046, Normoxic Control vs Normoxic GMP-ASC+ OA-SF

§ p= 0.017, Normoxic GMP-ASC +OA-CM vs Hypoxic GMP-ASC +OA-CM

^ p= 0.02, Hypoxic Control vs Hypoxic + GMP-ASC +OA-CM

# p= 0.046, Hypoxic Control vs Hypoxic GMP-ASC + OA-SF
